# miR-186-ANXA9 signaling inhibits tumorigenesis in breast cancer

**DOI:** 10.3389/fonc.2023.1166666

**Published:** 2023-09-29

**Authors:** Zhongrui Wang, Xiqian Zhou, Xiaochong Deng, Danrong Ye, Diya Liu, Baian Zhou, Wenfang Zheng, Xuehui Wang, Yuying Wang, Oyungerel Borkhuu, Lin Fang

**Affiliations:** ^1^ Department of Thyroid and Breast Surgery, Shanghai Tenth People’s Hospital, Shanghai Tenth People’s Hospital of Nanjing Medical University, Shanghai, China; ^2^ Department of Breast and Thyroid Surgery, Shanghai East Hospital, Tongji University School of Medicine, Shanghai, China; ^3^ Department of Breast and Thyroid Surgery, Shanghai Tenth People’s Hospital, Tongji University School of Medicine, Shanghai, China

**Keywords:** miR-186-5p, breast cancer, apoptosis, ANXA9, cell proliferation

## Abstract

Breast cancer (BC) ranks as the highest incidence among cancer types in women all over the world. MicroRNAs (miRNAs) are a class of short endogenous non-coding RNA in cells mostly functioning to silence the target mRNAs. In the current study, a miRNA screening analysis identified miR-186-5p to be downregulated in human breast cancer tumors. Functional studies *in vitro* demonstrated that overexpression of miR-186-5p inhibited cellular proliferation and induced cell apoptosis in multiple breast cancer cell lines including MDA-MB-231, MCF-7, and BT549 cells. Transplantation of the miR-186-5p-overexpressing MDA-MB-231 cells into nude mice significantly inhibited mammary tumor growth *in vivo*. Sequence blast analysis predicted annexin A9 (ANXA9) as a target gene of miR-186-5p, which was validated by luciferase reporter assay, QRT-PCR analysis, and western blot. Additional gene expression analysis of clinical tumor samples indicated a negative correlation between miR-186-5p and ANXA9 in human breast cancer. Knockdown of ANXA9 mimicked the phenotype of miR-186-5p overexpression. Reintroduction of ANXA9 back rescued the miR-186-5p-induced cell apoptosis. In addition, miR-186-5p decreased the expression of Bcl-2 and increased the expression of p53, suggesting a mechanism regulating miR-186-5p-induced cellular apoptosis. In summary, our study is the first to demonstrate miR-186-5p-ANXA9 signaling in suppressing human breast cancer. It provided a potential therapeutic target in breast cancer.

## Introduction

According to the latest cancer statistics report from WHO, breast cancer (BC) ranks as the highest incidence among cancer types in women all over the world (1). The number of new cases with BC is growing fast in women at a rate of around 19.9% in China (2). In the US, the estimated new cases of BC is 280,000 each year, accounting for 30% of all cancer cases in women (3). 12.9% of BC developed to invasive cancer according to the statistical data in the US (3). Pathogenesis of BC includes frequent gene mutations in BRCA1/2 (4) or TP53 (5), reduced DNA mismatch repair (MMR) (6), injury from radiation (7), and activation of carcinogenic signaling (8). In view of such a huge burden to women’s health with BC, it is urgently needed to explore the pathogenesis of BC and identify effective therapeutic targets in treatment of BC patients.

MicroRNAs (miRNAs) are a category of endogenous non-coding RNAs with 20–25 nucleotides in length (9). Mature miRNAs are usually assembled into RNA-induced silencing complexes (RISCs) (10), in which miRNAs recognize the 3′-UTR binding sites of target mRNAs through complementary base-pairing interaction (11) and thereby degrade target mRNA or repress mRNA translation according to the degree of complementarity (12). MiRNAs have been well demonstrated to regulate cell proliferation (13), differentiation (14), apoptosis (15), and cell cycle control (16). MiRNAs are also involved in the regulation of diverse human diseases including human cancer. For example, miR-5,096 was reported to inhibit cell migration by suppressing SLC7A11 and promote iron accumulation and ferroptosis in breast cancer (17). Overexpression of miR-9-3p was found to induce apoptosis-related gene expression in human breast cancer (18).

The annexin (ANX) family exists widely in nature (19), functioning as a calcium-regulating membrane binding protein (20, 21). ANX not only participates in the formation of cell membrane (22), regulating transmembrane transport (23) of calcium ion through endocytosis (24), but is also involved in regulation of the cell cycle (25) and cell apoptosis (26). So far, five families of ANX (ANX A–E) have been identified, in which only ANX A exists in vertebrates containing 12 family members from ANXA1 to ANXA13, excluding ANXA12 (19, 27). ANXA9 has been widely studied in tumors of the digestive system. Upregulation of ANXA9 was reported in gastric cancer, accelerating the malignant progression through regulating the TGF-β pathway (28). In addition, ANXA9 has been considered as a prognostic biomarker in colorectal cancer (29). Although exosome-derived ANXA9 was reported to function as an oncogene in breast cancer (30), the expression pattern and regulatory function of endogenous ANXA9 in breast cancer cells remain unclear.

Herein, we identified miR-186-5p with downregulation in breast cancer through a miRNA screening analysis of tumor samples. We demonstrated miR-186-5p as a tumor suppressor in breast cancer by targeting ANXA9. Overexpression of miR-186-5p induced cellular apoptosis, which was accompanied by downregulation of Bcl-2 and upregulation of p53. The current study demonstrated the miR-186-5p-ANXA9 signaling in suppressing human breast cancer.

## Materials and methods

### Human breast tumor samples

Human breast tumor samples were collected from Shanghai East Hospital. All the procedures were approved by the Institutional Review Board (IRB) of Shanghai East Hospital. All patients were provided with informed consent form.

### Animals

All procedures on the animal studies were approved by the Institutional Animal Care and Use Committee of the Tongji University School of Medicine. 8-week-old female nude mice were purchased from SiPeiFu (Beijing) Biotechnology Co., Ltd (Beijing, China).

### Vectors

The H149-CMV-EGFP-puro lentiviral vector was a product from Hanheng Biotechnology (Shanghai, China), which was applied to overexpress pre-miR-186-5p. The efficiency of miR-186-5p overexpression was determined by quantitative reverse transcription-polymerase chain reaction (QRT-PCR) analysis.

### Cell culture and transfection

Human breast cancer cells (MDA-MB-231, MCF-7, and BT-549 cells) were purchased form the cell bank of the Chinese Academy of Science (Shanghai, China). Human embryonic kidney 293 T cells (HEK293T) were a generous present from the Department of Central Lab at Shanghai Tenth People’s Hospital. MDA-MB-231, MCF-7, and HEK293T cells were cultured in Dulbecco’s modified Eagle’s medium (DMEM; Sigma, Merck, KGaA, Darmstadt, Germany) supplemented with 10% fetal bovine serum (FBS; Absin, Shanghai, China) and 1% penicillin–streptomycin (PS, 100 μg/ml; Beyotime, Shanghai, China). BT-549 cells were cultured in Roswell Park Memorial Institute 1640 (RPMI-1640; Gibco, Suzhou branch of Thermo Fisher Scientific, China), supplemented with 10% FBS and 1% PS. All of the cells were cultivated at 37°C in the incubator with sterilized air containing 5% CO_2_.

The oligo mimic of miR-186-5p and its negative control (NC) were synthesized by Shanghai Generay Biotech (Shanghai, China). Their sequences were miR-186-5p: sense, 5′-CAAAGAAUUCUCCUUUUGGGCU-3′, antisense: 5′-CCCAAAAGGAGAAUUCUUUGUU-3′; NC: sense, 5′-UCACAACCUCCUAGAAAGAGUAGA-3′, antisense: 5′-UACUCUUUCUAGGAGGUUGUGAUU-3′. The siRNA targeting ANXA9 (si-ANXA9) sequence 5′-CCCAACAGGACCUGAUGAAUU-3′ was synthesized by GenScript (Nanjing, China). si-NC was provided from GenScript. Lipo8000™ Transfection Reagent (Beyotime, Shanghai, China) was used for transfection of miR-186-5p mimic or si-ANXA9 following the manufacturers’ instruction.

### RNA extraction and quantitative reverse transcription-polymerase chain reaction

In 24–36 h after transfection, cells were collected and the total RNA was extracted by using EZ-press RNA Purification Kit (EZBioscience, Roseville, California, USA) following the manufacturers’ protocol. Reverse transcriptions were carried out using Hifair^®^ III 1st Strand cDNA Synthesis SuperMix (Yeasen, Shanghai, China) according to the manufacturers’ guidance. QRT-PCR was performed using a 7900 HT Fast Real-Time PCR System (7900HT Fast, Applied Biosystems, USA) and a QuantStudio Dx Real-Time PCR System (QuantStudio Dx, Applied Biosystems, USA). U6 was used to normalize miR-186-5p, and β-actin was used for mRNA normalization. The primers were synthesized by the Generay Biotech (Shanghai, China). Sequences of all primers were as follows (5′ to 3′), U6-F: GTGCTCGCTTCGGCAGCACATATAC, U6-R: AAAAATATGGAACGCTCACGAATTTG; 186-5p-F: CTCCAACGCAAAGAATTCTCC, 186-5p-R: TATGCTTGTTCTCGTCTCTGTGTC; β-actin-F: CATGTACGTTGCTATCCAGGC, β-actin-R: CTCCTTAATGTCACGCACGAT; ANXA9-F: CAGCTCATCTCACGAAACTTCC, ANXA9-R: GGTTCGAGTGGCAAGAATTTCAA. 2^−△△Ct^ formula was used to calculating the relative expression level of a miRNA or mRNA.

### Cell proliferation assay

Colony formation assay and methylthiazolyldiphenyl tetrazolium bromide (MTT) assay were used to determine the cellular proliferation ability. For colony formation assay, cells were resuspended and placed in six-well plates at a density of 800 cells per well. After 10–14 days when colonies formatted, they were fixed with 4% paraformaldehyde (Servicebio, Wuhan, China) and stained with 1% crystal violet (Yeasen, Shanghai, China). For MTT assay, cells were resuspended and placed in 96-well plates at a density of 2 × 10^3^ cells per well. MTT (Beyotime, Shanghai, China) reagent was dissolved in 1× PBS solution (Sangon Biotech, Shanghai, China) to reach a final concentration of 5 μg/μl. 20 µl of the MTT solution was added to each well at the indicated time points. After incubation for 4 h, the medium was replaced with 150 µl dimethyl sulfoxide (DMSO; Sangon Biotech, Shanghai, China) to dissolve formazan. SpectraMax^®^ iD5 (Molecular Devices, Sunnyvale, California, USA) was used to detect the absorbance at 490 nm.

### Cell apoptosis assay

Breast cancer cells were resuspended and seeded in six-well plates. In the indicated time after cell transfection, cells were trypsinized and collected into the flow tubes, followed by apoptotic analysis using FITC-Annexin V Apoptosis Detection Kit (eBioscience, Thermo Fisher Scientific, Massachusetts, USA) and flow cytometry (FACSCanto II, Becton, Dickinson and Company, New Jersey, USA).

### Cell cycle assay

Cells overexpressing miR-186-5p or NC were starved overnight in serum-free DMEM. After starvation, normal DMEM supplemented with 10% FBS was applied to proliferate cells for 12 h, followed by PI staining and cell cycle analysis (Beyotime, C1051) using flow cytometry (BD Biosciences, Mansfield, MA, USA).

### Dual-luciferase reporter assay

Two binding sites between miR-186-5p and ANXA9 mRNA were predicted by TargetScan (http://www.targetscan.org/vert_72/). psiCHECK-2-ANXA9 plasmids carrying either wild type or binding-site-mutated ANXA9 3′-UTR were purchased from IBSbio (Shanghai, China). HEK-293T cells and breast cancer cells were used for co-transfection with psiCHECK-2-ANXA9 and miR-186-5p mimic or NC. Dual-Luciferase Reporter Gene Assay Kit (Yeasen, Shanghai, China) was used to detect the luciferase activities of Firefly and Renilla.

### Western blot

Cellular lysates were prepared using RIPA buffer (Beyotime, Shanghai, China) supplemented with protease and phosphatase inhibitor cocktail (Beyotime, Shanghai, China). The protein concentration was quantified by BCA Protein Assay Kit (Beyotime, Shanghai, China). 30 µg protein lysates in each sample was loaded for sodium dodecyl sulfate polyacrylamide gel electrophoresis (SDS-PAGE) and subsequently transformed to nitrocellulose filter membranes (0.22 µm) (Pall, New York, USA) at constant current of 200 mA. 5% bull serum albumin (BSA; Sigma-Aldrich; Merck KGaA, Darmstadt, Germany) was used for blocking for 1 h at room temperature. Then, the membranes were incubated with corresponding primary antibodies overnight at 4°C, including ANXA9 (ab166621, Abcam, Cambridge, UK), Bcl-2 (#4223, CST, Massachusetts, USA), p-53 (10442-1-ap, Proteintech, Wuhan, China), and β-actin (AC026, ABclonal, Wuhan, China). After washing twice with Tris-HCl balanced salt buffer (TBS; Servicebio, Wuhan, China) containing 1% Tween (Sangon Biotech, Shanghai, China), anti-rabbit secondary fluorescent antibody (sc-2359; Santa Cruz Biotechnology, Santa Cruz, CA, USA) was used for incubation for 1 h at room temperature, followed by membrane scanning in the Odyssey CLx Infrared laser bicolor image analysis system (Li-Cor Biosciences, Nebraska, USA).

### *In vivo* tumor xenograft model

1.5 × 10^6^ of MDA-MB-231 cells overexpressing miR-186-5p were mixed with Matrigel and injected into the fat pat of the fourth mammary gland of female nude mice (n = 10). Tumor volumes were measured every 2 days until day 22 after cell transplantation when all the mice were euthanized.

### Immunohistochemistry staining

Tumor samples were fixed in 4% paraformaldehyde (Beyotime, China) and embedded in paraffin. The slides were incubated with ANXA9 primary antibody (sc-374288, Santa Cruz) at 4°C overnight, and then with HRP-linked Goat anti-Mouse IgG secondary antibody (ARG65350, Arigo) at room temperature for 1 h in the dark.

### Public database

The online website (https://www.xiantao.love/) was used to analyze TCGA dataset. The KM plotter (http://kmplot.com/analysis/) was used for survival curve analysis. miRNA–mRNA correlation analysis was performed using the Encyclopedia of RNA Interactomes (ENCORI, https://rnasysu.com/encori/index.php). The Human Protein Atlas (HPA, https://www.proteinatlas.org/) database was used for verification of ANXA9 protein levels in breast cancer tissues.

### Statistical analysis

All assays were repeated three times independently in triplicates. Images were generated by using GraphPad version 5.0 (GraphPad, California, USA). Statistical analyses were conducted by using SPSS 20.0 software (IBM, New York, USA). All the data were presented as the mean ± standard error (mean ± SEM) unless otherwise stated. Student’s t-test or two-way ANOVA test was used for significance test unless otherwise stated. Only p-values less than 0.05 were considered as statistically significant.

## Results

### Analyses of miR-186 in human breast cancer patients

**Figure 1:**
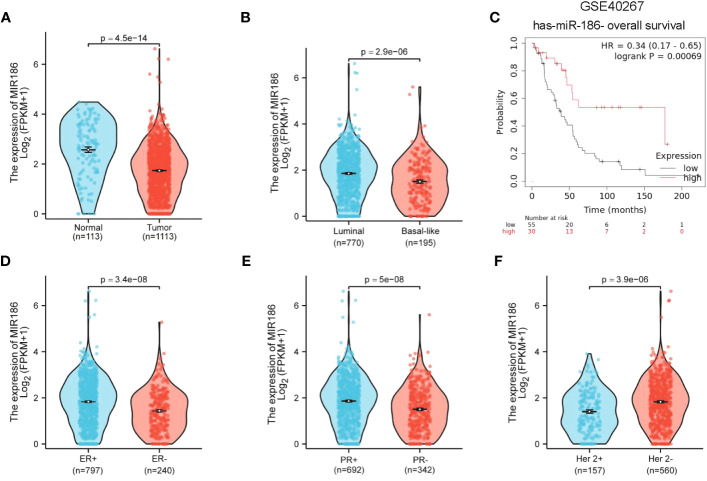
Downregulation of miR-186-5p in breast cancer. **(A)** Downregulation of miR-186-5p in breast cancer tissues (n = 1113), compared with normal controls (n = 113). **(B)** Lower levels of miR-186-5p in basal-like subtype (n = 195) of breast cancer than luminal subtype (n = 770). **(C)** A positive correlation between the expression level of miR-186-5p and overall survival in breast cancer patients. **(D–F)** Correlation analysis between the expression level of miR-186-5p and ER status **(D)**, PR status **(E)**, and Her 2 status **(F)** in breast cancer.

A miR expression analysis in human breast cancer using public datasets of TCGA identified miR-186 as an anti-oncomiRNA with downregulation in breast cancer tumors, compared with normal controls (**Figure 1A**). Moreover, miR-186 showed a lower level in the basal-like subtype of breast tumors, compared with luminal subtype (**Figure 1B**). KM plotter analysis indicated a correlation between the higher levels of miR-186 and better overall survival in breast cancer patients (**Figure 1C**). Notably, analysis of TCGA database showed an opposite correlation between miR-186 and overall survival, indicating the complexity of miR-186 in regulating human breast cancer, which may be dependent on the load of neoantigen (**Supplementary Figure S1**). Additional analyses indicated lower expression of miR-186 in the breast cancer patients with ER-negative (**Figure 1D**), PR-negative (**Figure 1E**), or HER-2-overexpression (**Figure 1F**). These results suggested a negative correlation between the expression level of miR-186 and the tumor aggressiveness in breast cancer.

### miR-186-5p suppressed breast cancer cell proliferation and arrested the cell cycle at the G_0_/G_1_ phase

**Figure 2:**
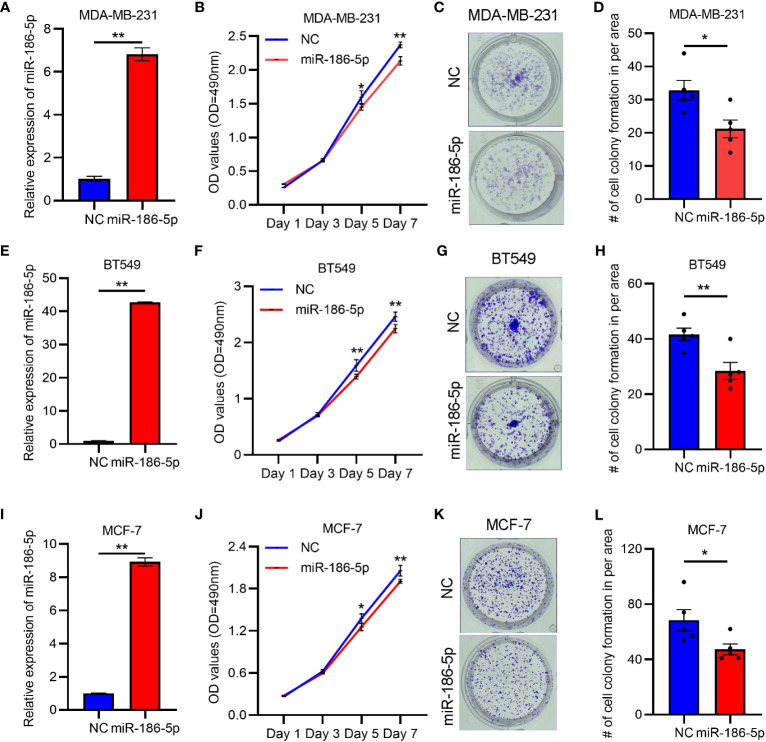
miR-186-5p inhibited cell proliferation in breast cancer. **(A)** Expressional analysis of miR-186-5p in MDA-MB-231 cells with or without transfection with miR-186-5p mimic. **(B)** MTT assays demonstrated miR-186-5p overexpression to inhibited cell proliferation in MDA-MB-231 cells. **(C)** miR-186-5p overexpression inhibited colony formation in MDA-MB-231 cells. **(D)** Quantitative analysis of the colonies in C. **(E–H)** Same assays as **(A–D)** were applied in BT549 cells with or without transfection with miR-186-5p mimic. **(I–L)** Same assays as **(A–D)** were applied in MCF-7 cells with or without transfection with miR-186-5p mimic. *p < 0.05, **p < 0.01 (n = 5).

In order to determine the biological function of miR-186-5p, we transfected the mimics of miR-186-5p into triple-negative breast cancer cell lines MDA-MB-231 and BT549, and luminal subtype cell line MCF-7 as well, followed by QRT-PCR analysis of miR-186-5p, MTT cell proliferation assay, and colony formation assay. As shown in **Figure 2A**, the transfection efficiency of miR-186-5p in MDA-MB-231 cells was confirmed. Overexpression of miR-186-5p in MDA-MB-231 cells significantly suppressed the cell proliferation (**Figure 2B**) and colony formation (**Figures 2C, D**). Similar results were observed in both BT549 (**Figures 2E–H**) and MCF-7 cells (**Figures 2I–L**). In addition, the cell cycle analysis was performed in MDA-MB-231 cells with or without overexpression of miR-186-5p. As shown in **Supplementary Figure S2**, miR-186-5p overexpression significantly arrested the cell cycle at the G_0_/G_1_ phase. Taken together, these results demonstrated suppression of breast cancer cell proliferation by miR-186-5p.

### miR-186-5p induced cellular apoptosis in breast cancer

**Figure 3:**
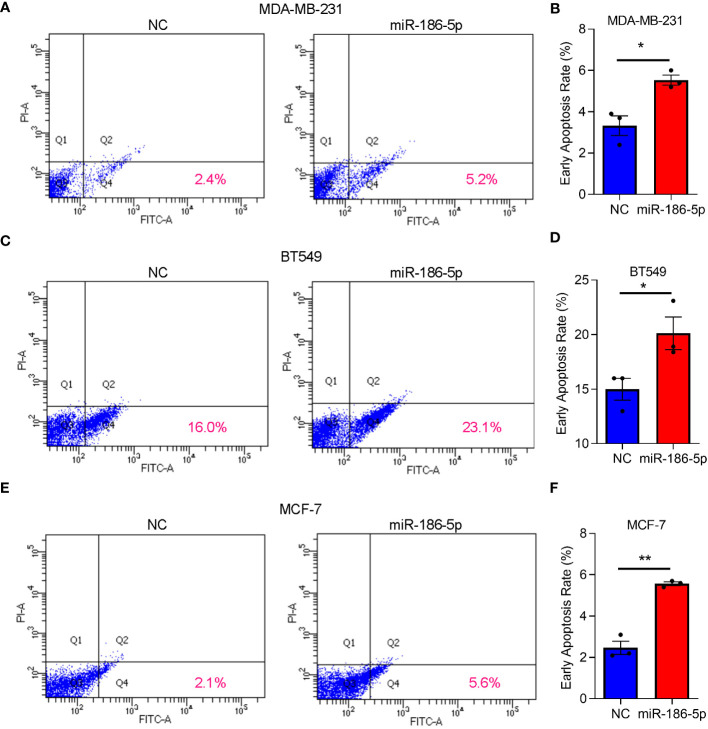
miR-186-5p induced cell apoptosis in breast cancer. **(A)** Cellular apoptosis analysis by flow cytometry in MDA-MB-231 cells with or without overexpression of miR-186-5p. **(B)** Quantitative analysis of the early apoptotic cells in A. **(C, D)** Same assays as **(A, B)** were applied in BT549 cells with or without overexpression of miR-186-5p. **(E, F)** Same assays as **(A, B)** were applied in MCF-7 cells with or without overexpression of miR-186-5p. *p < 0.05, **p < 0.01 (n = 3).

After transfection of miR-186-5p in MDA-MB-231, BT549, or MCF-7 cells, respectively, Annexin V staining and flow cytometry analysis were applied to determine the effects of miR-186-5p on cell apoptosis. As shown in **Figures 3A, B**, miR-186-5p overexpression induced apoptosis in MDA-MB-231 cells. Similar results were observed in both BT549 (**Figures 3C, D**) and MCF-7 cells (**Figures 3E, F**). A few proportion of cells in the NC group showed apoptosis due to either antibiotics in the cell culture medium or trypsin treatment during preparation of single-cell suspensions. However, much more cells showed early apoptosis in the miR-186-overexpressing cells in the same conditions with NC cells, demonstrating cell apoptotic induction by miR-186-5p.

### miR-186-5p suppressed tumor growth in a breast cancer mouse model

**Figure 4:**
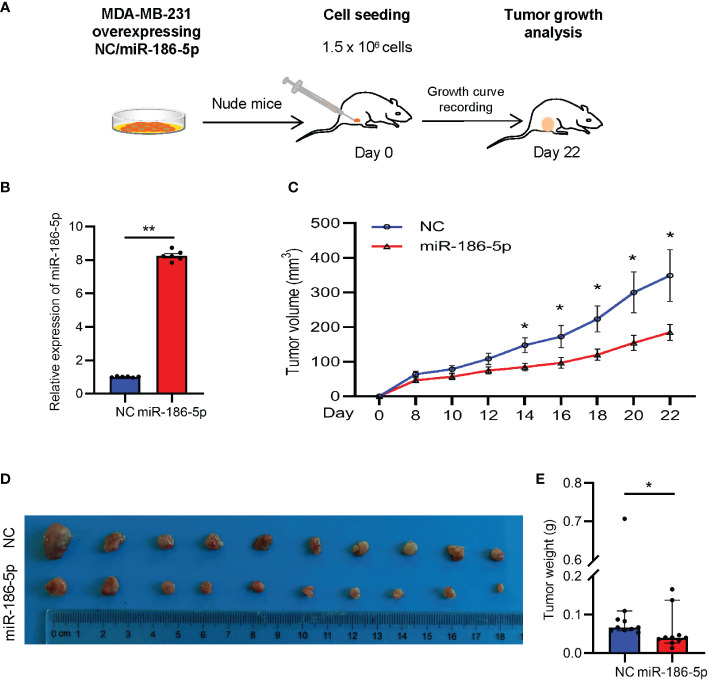
miR-186-5p suppressed tumor growth in a breast cancer mouse model. **(A)** Schematic procedure to prepare the xenograft mouse model with breast cancer. **(B)** Expressional analysis of miR-186-5p in MDA-MB-231 cells with or without stable infection of miR-186-5p. **(C)** Tumor growth curves showing decreased tumor growth rate by miR-186-5p overexpression *in vivo*. **(D)** Tumor Images from the mice in C. **(E)** Quantitative analysis of the tumor weight in **(D)** (n = 10 in each group). The statistical analysis method was Mann–Whitney test. The data were presented as median and 95%CI. *p < 0.05, **p < 0.01.

In order to determine the function of miR-186-5p in regulating tumorigenesis of breast cancer *in vivo*, the MDA-MB-231 cell line was selected to prepare the tumor xenograft model due to its strong tumorigenic capability. MDA-MB-231 cells stably overexpressing miR-186-5p or control were transplanted into the mammary fat pad of nude mice (n = 10 in each group), followed by tracking of the tumor volumes (**Figures 4A, B**). The tumor growth curves indicated a significant suppression of tumor growth by miR-186-5p (**Figure 4C**), which was further validated by the tumor size (**Figure 4D**) and tumor weight (**Figure 4E**) measured at day 22 with euthanasia of the mice.

### ANXA9 was identified as a direct target gene of miR-186-5p

**Figure 5:**
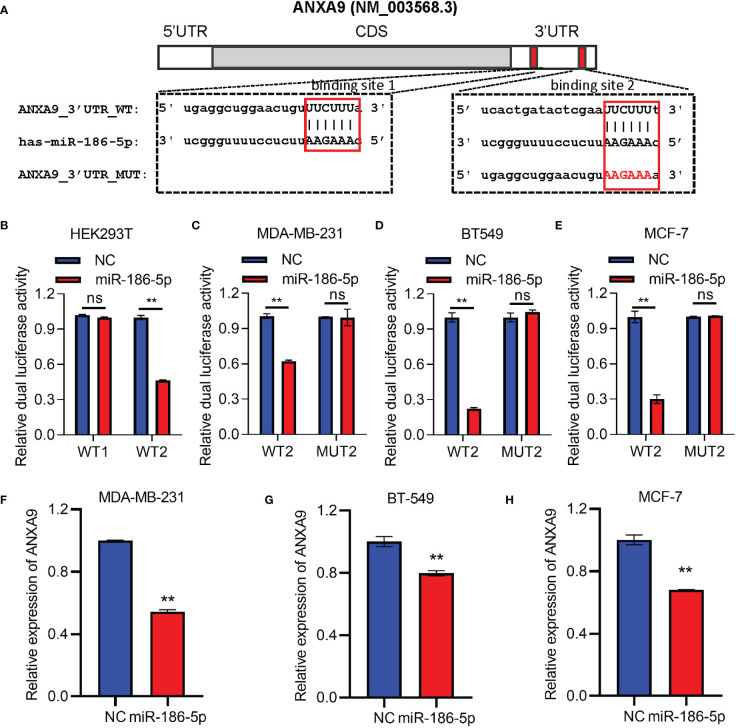
ANXA9 was identified as a target gene of miR-186-5p. **(A)** Sequence blast analysis indicated two binding sites to miR-186-5p in the 3′UTR of ANXA9 at nt145-nt151 (WT1) and nt299-nt305 (WT2). **(B)** Dual-luciferase reporter assays in HEK293T cells indicated inhibition of the WT2-reporter by miR-186-5p, but not WT1-reporter. **(C–E)** Dual-luciferase reporter assays in MDA-MB-231 **(C)**, BT549 **(D)**, or MCF-7 **(E)** cells indicated suppression of the WT2-reporter by miR-186-5p, not the MUT2-reporter (point mutations in the binding site 2. **(F–H)**. A QRT-PCR analysis demonstrated suppression of ANXA9 in expression by miR-186-5p in MDA-MB-231 **(F)**, BT549 **(G)** and MCF-7 **(H)** cells. **p < 0.01 (n=3). ns: non-significant.

A target prediction online tool TargetScan (http://www.targetscan.org/vert_72/) was applied to identify the target gene of miR-186-5p in breast cancer. As a result, ANXA9 was predicted as a potential target gene of miR-186-5p, which carries two binding sites at nt145 to nt151 and nt299 to nt305 with complementarity to miR-186-5p (**Figure 5A**). In order to demonstrate their direct target binding interaction, we first constructed two luciferase reporter plasmids carrying either an ANXA9 (WT1) fragment containing the first binding site to miR-186-5p or an ANXA9 (WT2) fragment containing the second binding site, followed by co-transfection with miR-186-5p mimic into HEK293T cells. The HEK293T cell line has been widely used as a research tool for gene reporter assays due to its high transfection efficiency and improved expression of exogenous genes. Before performing luciferase reporter assays, we analyzed the basal expression of miR-186-5p in HEK293T cells using a public dataset GSE214609. As shown in **Supplemental Figure S3**, endogenous miR-186-5p showed much less amount of reads than reference gene miR-16-5p, indicating its low expression level in HER293T cells.

Luciferase activity analysis showed inhibition of the ANXA9 WT2-reporter by miR-186-5p, but not ANXA9 WT1-reporter (**Figure 5B**), suggesting that the target interaction between miR-186-5p and ANXA9 was mediated by binding site 2, but not binding site 1. In order to further confirm the target interaction, point mutations were applied to binding site 2 (MUT2). Breast cancer cells MDA-MB-231, BT549, or MCF-7 were co-transfected with miR-186-5p mimic and WT2- or MUT2- reporter, respectively. As shown in **Figures 5C–E**, miR-186-5p suppressed the WT2-reporter activity in all the three breast cancer cell lines, which were attenuated by the point mutations (MUT2-reporter). Moreover, our analysis of the ANXA9 mRNA levels by QRT-PCR further demonstrated suppression of ANXA9 in expression by miR-186-5p in breast cancer cells (**Figures 5F–H**).

### Clinical relevance of miR-186-5p and ANXA9 in human breast cancer tumors

**Figure 6:**
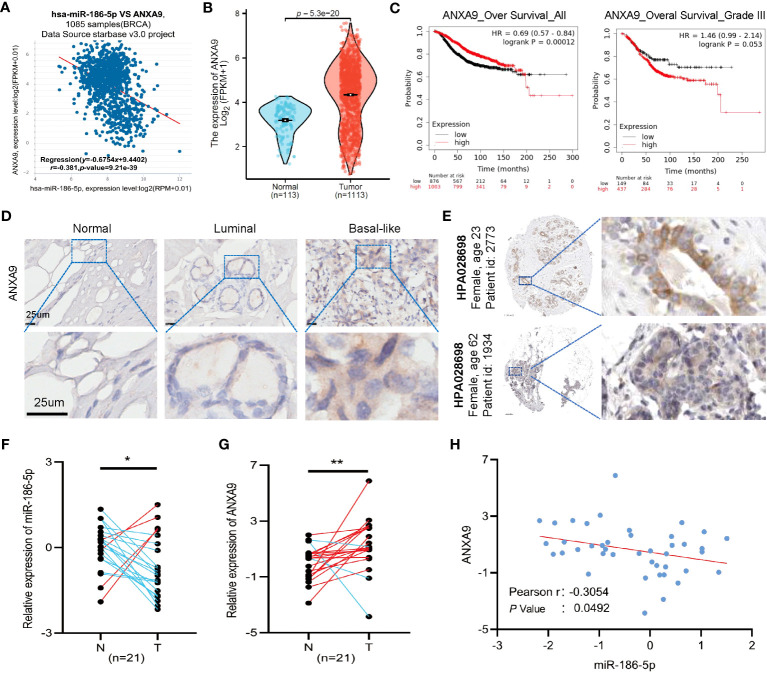
Clinical relevance of miR-186-5p and ANXA9 in human breast cancer tumors. **(A)** Negative correlation between ANXA9 and miR-186-5p in expression in 1085 breast cancer patients. **(B)** Aberrant upregulation of ANXA9 in human breast cancer tumors (n = 1113), compared with normal controls (n = 113). **(C)** Kaplan–Meier plotter analysis showed that higher expression of ANXA9 was correlated with shorter survival in BC patients in long-term or with more aggressive grade III. **(D)** Immunohistochemical (IHC) staining of ANXA9 in luminal and basal-like subtypes of human breast cancer tumors (n = 3 for each), compared with normal control tissues. **(E)** IHC staining of ANXA9 in additional breast tumor samples from the Human Protein Atlas database. **(F, G)** Expression analysis of miR-186-5p **(F)** and ANXA9 **(G)** in 21 tumor samples and matching adjacent normal tissues from human breast cancer patients. **(H)** A negative correlation between miR-186-5p and ANXA9 in the 21 tumor samples from human breast cancer patients. *p < 0.05, **p < 0.01 (n=21).

Since ANXA9 was identified as a target gene of miR-186-5p, we analyzed the expression pattern of ANXA9 in breast cancer patients. A negative correlation between the expression of ANXA9 and the miR-186-5p level was demonstrated by applying ENCORI analysis (http://starbase.sysu.edu.cn/index.php) of a public dataset including in 1085 breast cancer patients (**Figure 6A**). Additional analysis of TCGA database indicated aberrant upregulation of ANXA9 in human breast cancer tumors (**Figure 6B**). Kaplan–Meier plotter analysis showed that a higher expression of ANXA9 was correlated with shorter survival in BC patients in the long term or with more aggressive grade III (**Figure 6C**). Notably, an opposite correlation was observed in BC patients with less aggressive grades I and II (**Supplementary Figure S4**), suggesting that ANXA9 regulation of breast cancer may be related with the tumor aggressiveness and cancer progression.

In addition, we applied immunohistochemical (IHC) staining of ANXA9 in both luminal and basal-like subtypes of human breast cancer tumors (n = 3 for each), compared with normal controls. As seen in **Figure 6D**, ANXA9 showed obvious upregulation in both subtypes of breast cancer, which was further validated by additional breast tumor samples from the Human Protein Atlas database (**Figure 6E**). Moreover, we detected the mRNA levels of ANXA9 and miR-186-5p in 21 tumor tissues and matching adjacent normal tissues from human breast cancer patients. As shown in **Figures 6F, G**, the expression of miR-186-5p showed significant downregulation, whereas ANXA9 showed upregulation in breast tumor tissues, compared with normal controls. A negative correlation between miR-186-5p and ANXA9 further confirmed the negative regulation of ANXA9 by miR-186-5p (**Figure 6H**).

### ANXA9 overexpression rescued the effect of miR-186-5p in breast cancer cells

**Figure 7:**
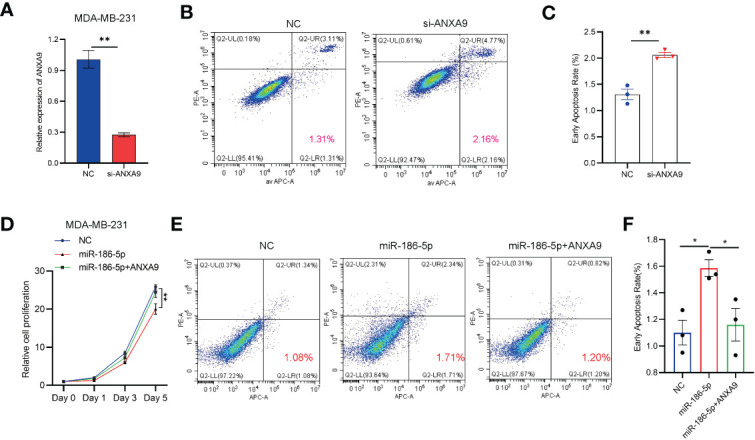
ANXA9 overexpression rescued the effect of miR-186-5p in breast cancer cells. **(A)** Knocked down ANXA9 by si-ANXA9 in MDA-MB-231 cells. **(B)** Cell apoptotic analysis of MDA-MB-231 cells with or without knockdown of ANXA9. **(C)** Quantitative analysis of the early apoptotic cells in B. **(D)** CCK8 cell proliferation assays in MDA-MB-231 cells overexpressing miR-186-5p with or without cotransfection of pcDNA3.1-ANXA9. **(E)** Cell apoptosis analysis of MDA-MB-231 cells overexpressing miR-186-5p with or without cotransfection of pcDNA3.1-ANXA9. **(F)** Quantitative analysis of the early apoptotic cells in **(E)** *p < 0.05, **p < 0.01 (n = 3).

In order to further demonstrate miR-186-5p induction of cell apoptosis by inhibiting ANXA9 in breast cancer, we knocked down ANXA9 by si-ANXA9 in MDA-MB-231 cells to mimic the overexpression of miR-186-5p (**Figure 7A**), followed by the cell apoptotic analysis. As shown in **Figures 7B, C**, ANXA9 knockdown induced apoptosis in MDA-MB-231 cells. Moreover, we prepared a pcDNA3.1-based plasmid carrying cDNA encoding ANXA9. MDA-MB-231 cells was co-transfected with pcDNA3.1-ANXA9 and miR-186-5p, followed by CCK8 assay and cell apoptosis analysis. As shown in **Figures 7D–F**, overexpression of ANXA9 rescued the cell proliferation (**Figure 7D**) and reversed the miR-186-5p-induced cell apoptosis (**Figures 7E, F**).

### miR-186-5p suppressed Bcl-2 and promoted p53 in expression in breast cancer

**Figure 8:**
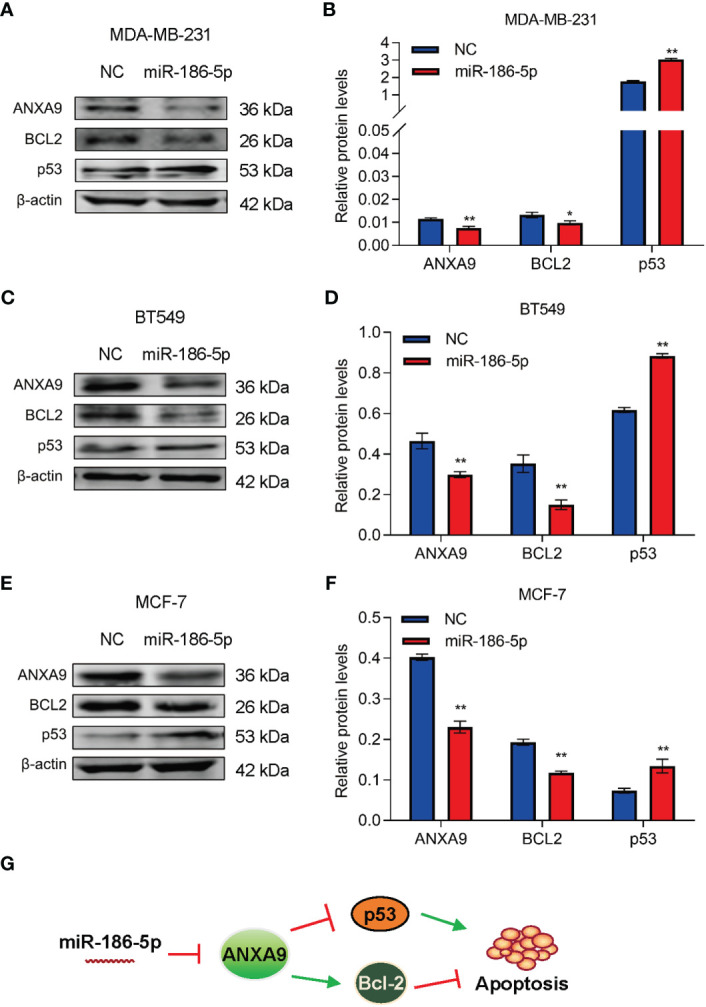
miR-186-5p suppressed Bcl-2 and promoted p53 in breast cancer. **(A)** Western blot analysis indicated downregulation of ANXA9 and Bcl-2, while upregulation of p53 in the miR-186-5p-overexpressing MDA-MB-231 cells. **(B)** Quantitative analysis of A. **(C, D)** Same assays as A-B were applied in BT549 cells with or without overexpression of miR-186-5p. **(E, F)** Same assays as A-B were applied in MCF-7 cells with or without overexpression of miR-186-5p. **(G)** Schematic representation of the working model in which Bcl-2 and p53 involve in the miR-186-5p induced cell apoptosis. *p < 0.05, **p < 0.01.

In view of the literature reports of miR-186-5p involvement in regulation of cell apoptosis (31–33), we detected two of the most important cell apoptotic regulators, Bcl-2 and p53, in breast cancer cells by western blot analysis. Downregulation of Bcl-2 and upregulation of p53 were associated with inhibition of ANXA9 in the miR-186-5p-overexpressing MDA-MB-231 cells (**Figures 8A, B**; **Supplementary Figure S8**). Similar results were observed in both BT549 (**Figures 8C, D**; **Supplementary Figure S9**) and MCF-7 cells (**Figures 8E, F**; **Supplementary Figure S10**). We further analyzed the relationships between miR-186-5p, ANXA9, and BCL2 in breast cancer tumors by performing the ENCORI analysis of a public dataset. As expected, a positive correlation between the expression levels of ANXA9 and BCL2 was observed in breast cancer patients (n = 1104, **Supplementary Figure S5A**), as well as a negative correlation between miR-186-5p and BCL2 (n = 1085, **Supplementary Figure S5B**). These results suggested that both Bcl-2 signaling and p53 signaling were involved in the miR-186-5p-ANXA9-induced apoptosis in breast cancer (**Figure 8G**).

Additionally, we analyzed the expression of apoptosis-related gene caspase 3. As shown in **Supplemental Figure S6A**, caspase 3 showed upregulation by miR-186-5p in MDA-MB-231 cells. A positive relationship between miR-186-5p and caspase 3 was confirmed in 1085 breast cancer samples (n = 1085, **Supplementary Figure S6B**).

## Discussion

The incidence of breast cancer is still increasing in women all over the world (34), which is mainly due to abnormal expression of oncogenes or tumor-suppressor genes (35–37). However, the overall survival of patients with breast cancer has been greatly prolonged since the widespread of physical screening examination and improvement of therapeutic strategy (38).

MiRNAs have been well demonstrated to regulate diverse types of human cancer (39). miR-186-5p was reported to be a tumor suppressor in various malignant tumors including bladder cancer (40), colorectal cancer (33), osteosarcoma (41), and breast cancer (42). Overexpression of miR-186-5p inhibited cellular proliferation, migration, and invasion in non-small-cell lung cancer cells and promoted sensitivity to paclitaxel by binding to sine oculis homeobox 1 (SIX1) (32). In hepatocellular carcinoma (HCC), miR-186-5p interacted with long non-coding RNA 665 (LINC00665) and circular RNA protein kinase C iota (circ-PRKCI) to inhibit cellular viability and tumor progression by regulating Forkhead Box K1 (FOXK1) expression. In addition, miR-186-5p induced apoptosis and autophagy in HCC through binding mitogen-activated protein kinase 3 (MAP4K3) (43, 44). However, the expression pattern and biological function of miR-186-5p in breast cancer remain unclear.

In the current study, overexpression of miR-186-5p inhibited breast cancer cellular proliferation *in vitro* and mammary tumor growth *in vivo* and induced the cellular apoptosis by regulating Bcl-2 and p53 signaling. In mechanism, ANXA9 was identified as a target gene of miR-186-5p. The clinical relevance of miR-186-5p-ANXA9 was further confirmed by analysis of patients with breast cancer.

According to the official website of miRNAs, miRBase, miR-186-5p is the dominant arm of miR-186 in human cells (**Supplemental Figure S7**). Consistently, herein we successfully detected the expression of miR-186-5p in breast cancer patients and demonstrated downregulation of miR-186-5p in the breast tumors (**Figure 6**), but miR-186-3p was undetectable in the same samples.

According to TargetScanHuman 8.0 (www.targetscan.org), ANXA9 has two binding sites to miR-186-5p. The first binding site is located at position 145–151 of ANXA9 3′ UTR, which is poorly conserved among mammals. The second binding site is located at the position 299–305 of ANXA9 3′ UTR, which is conserved among human, pig, rabbit, cat, dog, etc. Our luciferase reporter assays demonstrated the interaction between ANXA9 and miR-186-5p via the second binding site, but not the first binding site (**Figure 5**).

ANX, as a calcium-dependent membrane binding protein, exists widely from mammals to prokaryotes, regulating tissue development and various biological functions. It was first discovered in 1977 and characterized with soluble, thermally unstable, and activated upon calcium ions (45–47). Abnormal expression of ANX is frequently reported to regulate apoptosis in human malignant tumor cells. For example, ANXA1 was reported to regulate glioma cell apoptosis (48). ANXA2, as a downstream gene of bufalin, was reported to interact with mitochondria, leading to disruption of the mitochondrial division/fusion balance and induction of glioma cell apoptosis (26). ANXA9 was reported to regulate cell proliferation and migration in gastric cancer (28). Our current study demonstrated miR-186-5p to increase the expression of p53 while decrease the expression of Bcl-2, two key regulators of cell apoptosis (49). These findings suggest miR-186-5p-ANXA9 may involve in the regulation of cell apoptosis in breast cancer.

According to the literature, miR-105-1/ANXA9 axis played an important role in regulating cisplatin-induced cell apoptosis in ovarian cancer (50), in which the cell apoptotic regulators Bcl-2 and p53 were involved. Since ANXA9 was identified as a target gene of miR-186-5p in the current study, we analyzed the relationships between miR-186-5p/ANXA9 and Bcl-2 or p53. Our results (**Figure 8**) suggested that Bcl-2 and p53 may be involved in the miR-186-5p-ANXA9-induced apoptosis in breast cancer. More experimental analyses will be required for further validation.

In summary, this study demonstrated the tumor-suppression function of miR-186-5p in human breast cancer. It is the first to show the miR-186-5p/-NXA9 signaling in regulating cancer cell apoptosis, providing a novel therapeutic target in breast cancer.

## Data availability statement

The original contributions presented in the study are included in the article/**Supplementary Material**. Further inquiries can be directed to the corresponding author.

## Ethics statement

The studies involving humans were approved by the Institutional Review Board (IRB) of Shanghai East Hospital. The studies were conducted in accordance with the local legislation and institutional requirements. The participants provided their written informed consent to participate in this study. The animal study was approved by the Institutional Animal Care and Use Committee of the Tongji University School of Medicine. The study was conducted in accordance with the local legislation and institutional requirements.

## Author contributions

LF designed the study and supervised all experiments. ZW and XZ performed experiments and wrote the manuscript. XD, DY, DL, and BZ helped to check clinical database and made the related figures. WZ and XW helped conduct cell flow cytometry assays. YW and OB advised dual-luciferase assays. All authors contributed to the article and approved the submitted version.
