# Supplementary material for: miR-186-ANXA9 signaling inhibits tumorigenesis in breast cancer
Source: Front Oncol. 2023 Sep 29;13:1166666. doi: 10.3389/fonc.2023.1166666 (PMC10570552; doi:10.3389/fonc.2023.1166666)

Supplemental Figure legends

Figure S1. Correlation analysis between miR-186 and overall survival in breast cancer patients using the TCGA dataset showed a Neoantigen load-dependent pattern.

Figure S2. Cell cycle analysis in MDA-MB-231 cells with or without overexpression of miR-186-5p.

(A) The cell cycly analysis; (B) Quantitative analysis of the cells at G_0/G_1 phase and the cells at S phase in A. *$p < 0.05$ (n=3).

Figure S3. Basal expression of endogenous miR-186-5p and miR-186-3p in HEK293T cells using a public dataset GSE214609. miR-16-5p was used as a reference gene for comparison.

Figure S4. Higher expression of ANXA9 was correlated with longer survival in BC patients with less aggressive grades I and II.

Figure S5. Relationships between miR-186-5p, ANXA9 and BCL2 in breast cancer tumors. (A) A positive correlation between the expression levels of ANXA9 and BCL2 in breast cancer patients (n=1,104). (B) A negative correlation between the expression levels of miR-186-5p and BCL2 in breast cancer patients (n=1,085).

Figure S6. (A) Analysis of apoptosis-related gene caspase 3 in MDA-MB-231 cells with or without overexpression of miR-186-5p. (B) A positive correlation between miR-186-5p and caspase 3 in 1,085 breast cancer samples.

Figure S7. miRBase showing miR-186-5p as the dominant arm of miR-186 in human cells.

Figure S8. Original uncropped images for Figure 8A.

Figure S9. Original uncropped images for Figure 8C.

Figure S10. Original uncropped images for Figure8E.

Supplementary Figure S1

TCGA: Neoantigen load: high

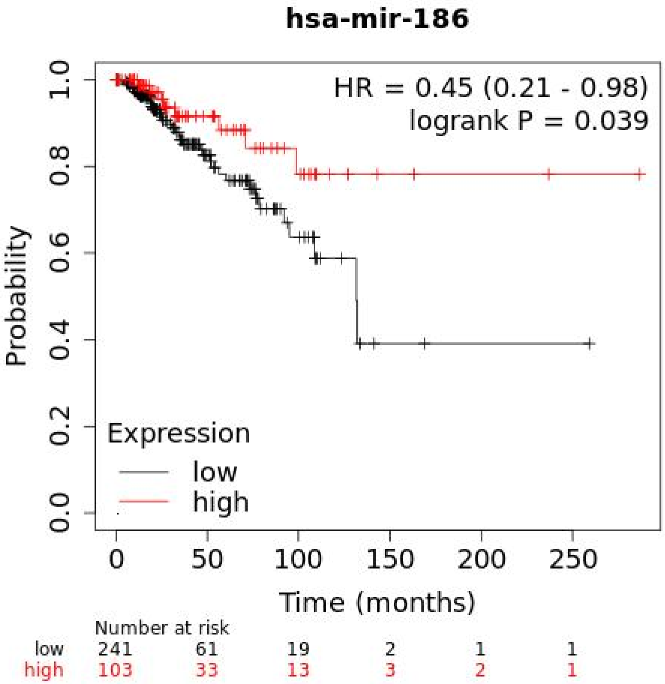

TCGA: Neoantigen load: low

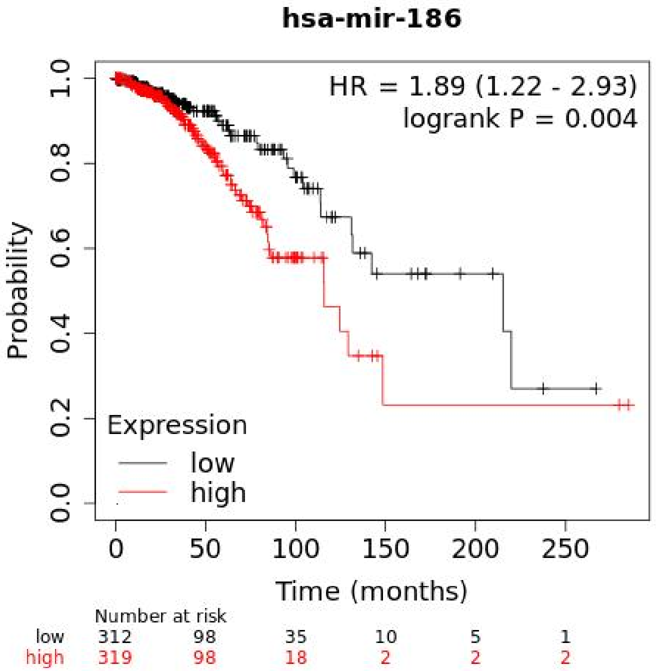

Supplementary Figure S2

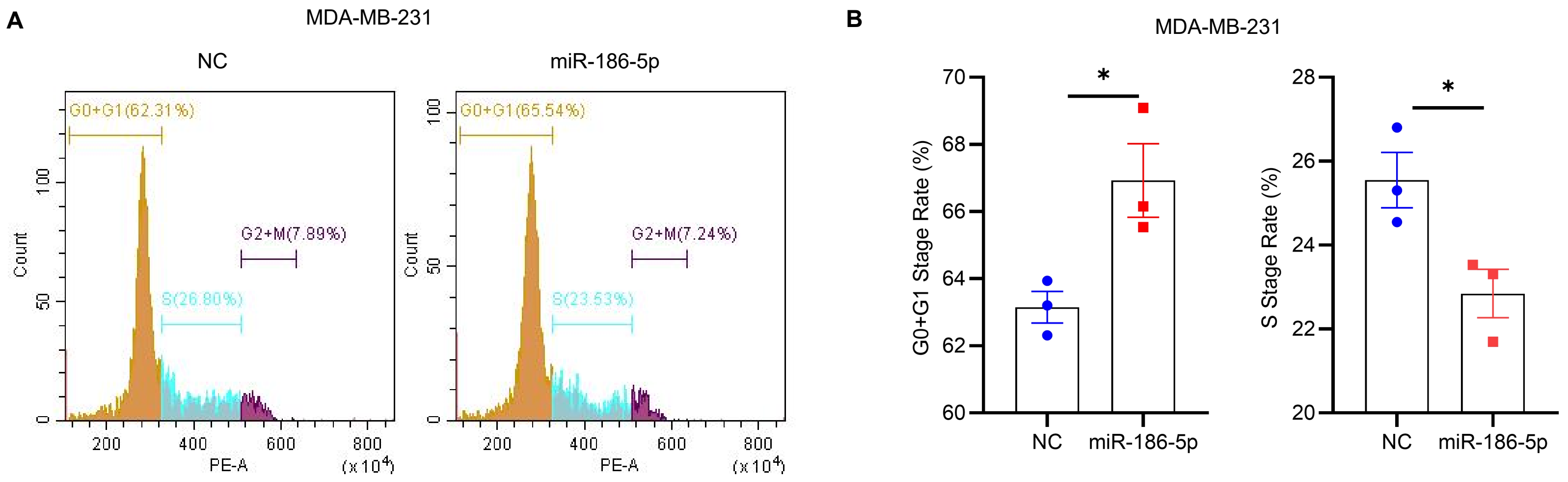

Supplementary Figure S3

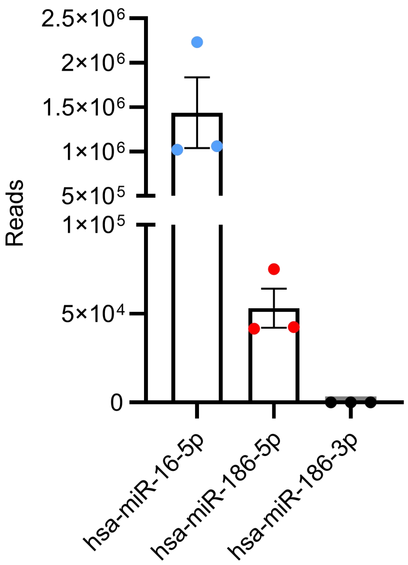

Supplementary Figure S4

ANXA9_Overal Survival
Grade II

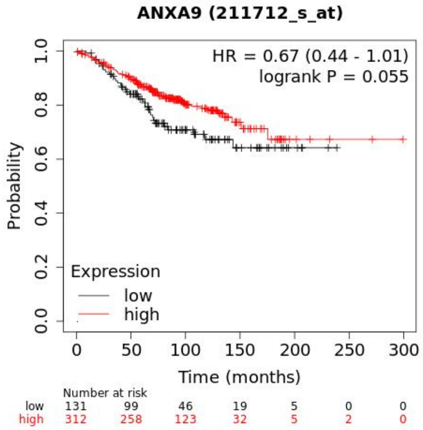

ANXA9_Overal Survival
Grade I

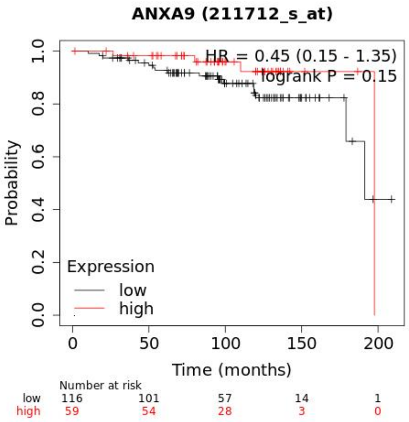

Supplementary Figure S5

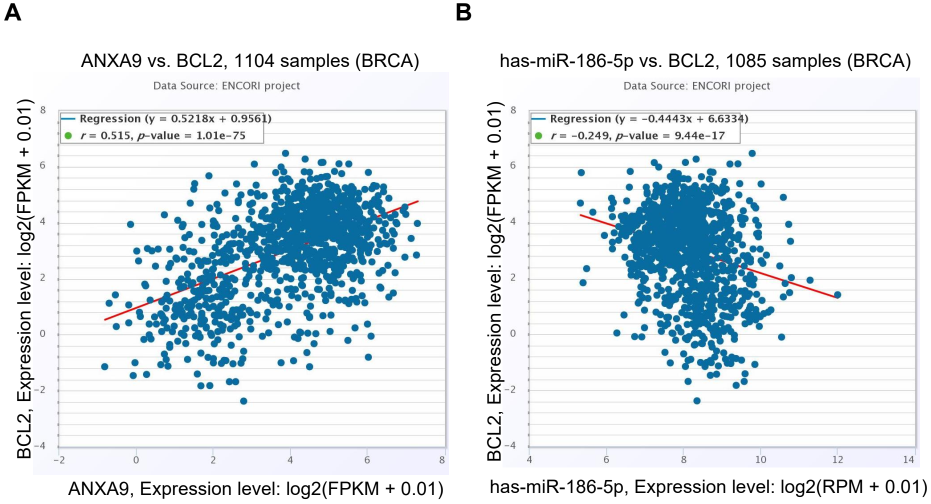

Supplementary Figure S6

A

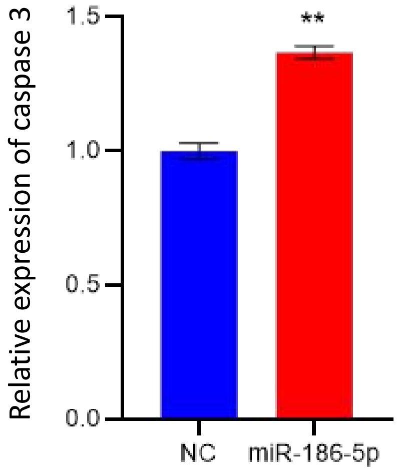

B

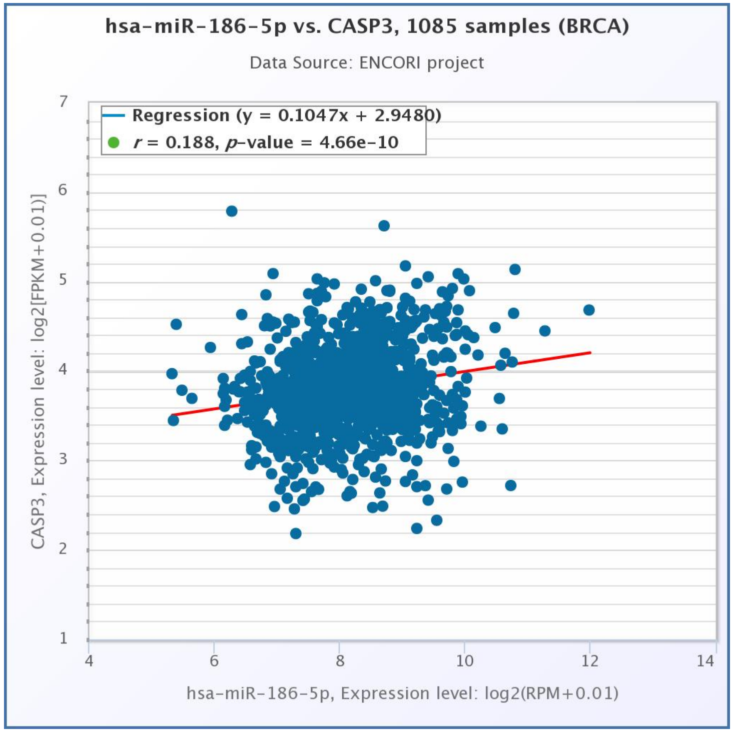

Supplementary Figure S7

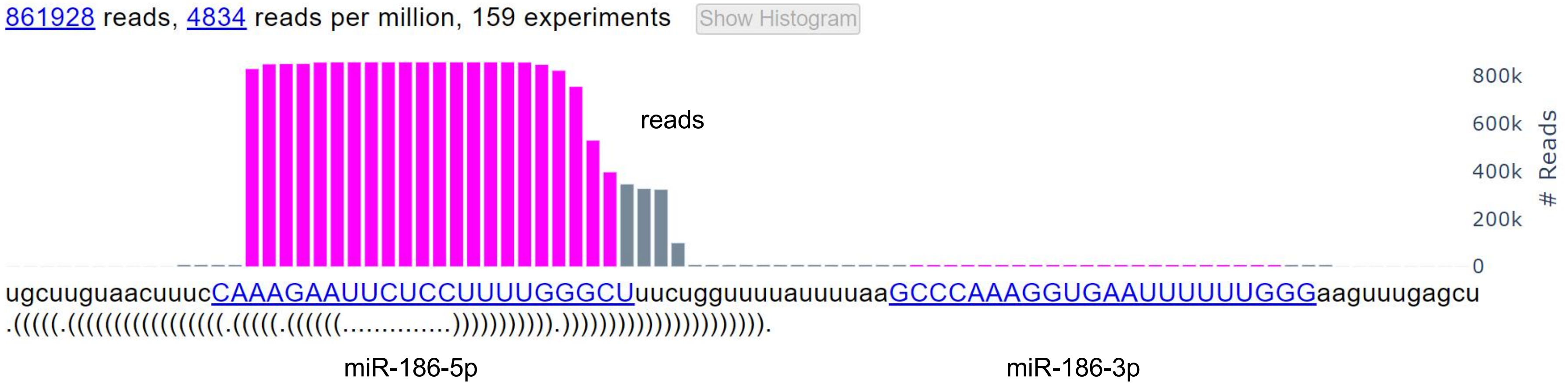

Supplementary Figure S8 Original uncropped images for Figure 8A

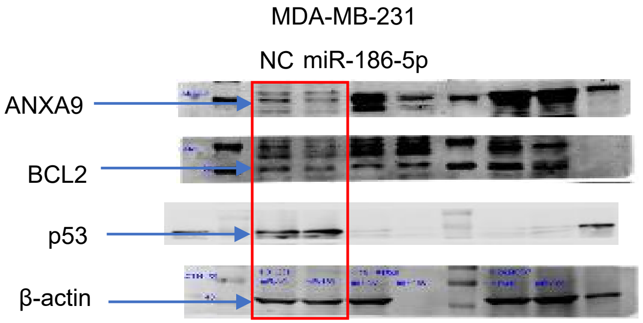

Supplementary Figure S9 Original uncropped images for Figure 8C

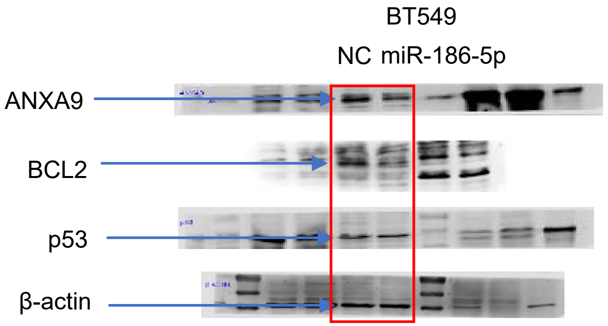

Supplementary Figure S10 Original uncropped images for Figure 8E

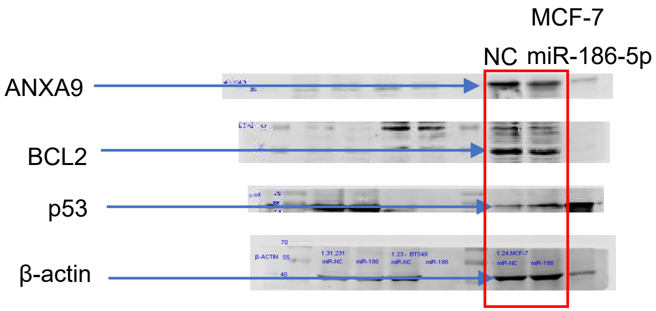

Supplement: Supplementary file 1 [file DataSheet_1.pdf]
